# A modified data normalization method for GC-MS-based metabolomics to minimize batch variation

**DOI:** 10.1186/2193-1801-3-439

**Published:** 2014-08-19

**Authors:** Mingjie Chen, R Shyama Prasad Rao, Yiming Zhang, Cathy Xiaoyan Zhong, Jay J Thelen

**Affiliations:** Department of Biochemistry, Interdisciplinary Plant Group, Christopher S. Bond Life Science Center, University of Missouri, Columbia, MO 65211 USA; Regulatory Science, DuPont Experimental Station, Route 141 and Henry Clay Road, Delaware, 19880 USA

**Keywords:** Maize, Batch-to-batch variation, Metabolomics, Normalization, Reference sample

## Abstract

The goal of metabolomics data pre-processing is to eliminate systematic variation, such that biologically-related metabolite signatures are detected by statistical pattern recognition. Although several methods have been developed to tackle the issue of batch-to-batch variation, each method has its advantages and disadvantages. In this study, we used a reference sample as a normalization standard for test samples within the same batch, and each metabolite value is expressed as a ratio relative to its counterpart in the reference sample. We then applied this approach to a large multi-batch data set to facilitate intra- and inter-batch data integration. Our results demonstrate that normalization to a single reference standard has the potential to minimize batch-to-batch data variation across a large, multi-batch data set.

**Electronic supplementary material:**

The online version of this article (doi:10.1186/2193-1801-3-439) contains supplementary material, which is available to authorized users.

## Introduction

Success of metabolomics as a phenotyping or diagnostic platform depends on its ability to detect biologically-related global metabolite changes in complex biological samples. The variability in samples can arise from multiple sources including physiological differences and variability from the analytical method itself. Removing platform-specific sources of variability such as systematic error is one of the top priorities in metabolomics data preprocessing. However, metabolite diversity leads to different responses to variations at given experimental conditions, making normalization a very demanding task (Sysi-Aho et al. 2007).

Batch-to-batch variation is a technical source of variation arising from the sum of all sample handling (both manual and robotic) steps and has been documented in large-scale NMR and LC-MS metabolomic studies (Teahan et al. 2006; Tate et al. 2001; Wagner et al. 2007). For example, MS performance changes as columns are liable to degradation over time (Sangster et al. 2006). The presence of batch-to-batch variation makes it difficult to integrate inter-batch data (Wagner et al. 2007). To eliminate batch-to-batch variation, several normalization methods have been developed including: scalar correction (Crawford and Morrison 1968; Wang et al. 2003); internal standard (ISTD, Sysi-Aho et al. 2007; Redestig et al. 2009); quality control or reference sample (Bolstad et al. 2003; Sangster et al. 2006; Jauhiainen et al. 2014; Dunn et al. 2011) and variance-based normalization (De Livera et al. 2012). The scalar normalization method does not use internal standards, and normalizes to a total sum or the median of each sample. This method assumes equivalent total metabolite signal or equivalent mean/median value per sample. This method is suitable when the majority of all analytes remain constant. However, this ideal situation typically does not hold due to the nature of the samples, therefore such normalization may distort data potentially masking true biological trends.

The isotope-labeled internal standard (IS) approach was used to monitor the analytical error. The IS is a known metabolite with a defined quantity. Expressing analyte abundances relative to the IS can suppress technical errors. The assumption underlying this method is that variation in IS can only result from systematic errors, and different analytes behave similar to IS. When isotope labeled metabolites do not co-elute with the unlabeled version, changes in the concentration of one compound can cause variance in the measurements of a different compound due to insufficient HPLC chromatographic separation or ion suppression in LC-MS (Liu et al. 2002; Annesley 2003), commonly referred to as matrix effect. Furthermore, the quantitation of metabolites could also suffer from difference in derivatization efficiency of internal standard for GC-MS platform.

Since it is difficult to use any single IS to get a reliable estimation of the systematic error on a complex metabolite mixture the NOMIS method (Normalization using Optimal selection of Multiple Internal Standards) was developed (Bijlsma et al. 2006; Katajamaa et al. 2006; Sysi-Aho et al. 2007). However, the application of multiple ISs has generated another problem called cross-contribution (CC), in which analytes may directly influence estimates of the IS (Liu et al. 2002; Redestig et al. 2009). Several correction algorithms have been developed to eliminate systematic CC effects (Deport et al. 2006; Engel and Ratel 2007; Sysi-Aho et al. 2007; Redestig et al. 2009). Each algorithm removes, to varying degrees of proficiency, run order and batch effects without losing informative variance.

Quality control (QC) samples that are generally used to assess the performance of the system are now used for calibration purposes. Mean and median correction was used to account for the batch to batch variation (Kloet et al. 2009). In this method the QC samples were used to calculate batch/analyte specific correction factor by dividing batch median to global median, then apply correction factor to test samples. This method requires many quality control samples, is very susceptible to outliers, and can inflate variance when training and test set trends do not match. A second method was called quality control-based robust LOESS signal correction (QC-RLSC) (Dunn et al. 2011). In this method one cannot assume convergence of training and test sample performance because test samples have both analytical and biological variance. This normalization method can inflate variance when overtrained or training examples do not match the test set. Besides these normalization methods, a variance based method also was developed to remove unwanted batch variation (De Livera, et al., 2012).

To simplify the inter-batch data integration process, we inserted a reference sample within each batch and run several technical replicates during the course of batch analysis. Statistical analysis of data obtained from the reference samples from dozens of batches demonstrated that batch-to-batch variation is an important source of systematic variation in GC-MS analysis. To eliminate batch effects, we also used a reference sample as a normalization standard for test samples and express analyte content in the test sample as a ratio relative to its counterpart in the reference sample. Our results demonstrate that this normalization method can minimize the batch-to-batch data variability across extended periods required for large-scale phenotyping and facilitate inter-batch data integration.

## Materials and methods

### Plant materials

Fifty non-GM maize hybrid entries from DuPont Pioneer were grown at six locations (Texas, Kansas, Illinois, Nebraska, Minnesota and Ontario), a maximum of 20 entries were grown in each site. The block design, planting, and sample harvesting were detailed previously (Asiago et al. 2012). Forage samples representing the pooled portions of three entire plants after flowering were collected for each genotype and block.

### Polar metabolites extraction and derivatization

Metabolites were extracted from dry ground powder of forage samples. For each forage sample, 2.5-3.5 mg was weighed and transferred into a 2-mL microfuge tube to which 0.75 mL of chloroform (Fisher Scientific, New Jersey) was added. Samples were incubated at 55°C with rotation for 30 min, then 0.75 mL of deionized water (18 MΩ) containing 5 μg/mL ribitol (TCI, Portland, OR) was added and incubated for an additional 30 min. Samples were then centrifuged at 1500 g for 15 min to allow phase separation. Six hundred and sixty microliters of the upper aqueous phase were carefully transferred into a 2-mL glass GC vial and subsequently evaporated to dryness in a speed vac. Before GC-MS analysis, test samples from the same site were arranged into batches (Additional file 1: Table S1). One reference sample was included within each batch. The forage reference samples were obtained from Illinois site. Plant tissue for the reference sample entries was pooled and mixed thoroughly, and metabolites were extracted as test samples.

The dried extracts were dissolved in 120 μL of 20 mg/mL methoxyamine hydrochloride (Sigma-Aldrich, Switzerland) in pyridine and incubated at 37°C for 90 min to form methoxyamine derivatives. Subsequently, 120 μL of N-methyl-N-(trimethylsiyl) trifluoroacetamide (MSTFA, Thermo Scientific, PA, US) plus 1%TMCS were added and the extracts were incubated at 37°C for 90 min to form trimethylsilyl derivatives.

### GC-MS analysis

Derivatized metabolite mixtures were analyzed by a Hewlett Packard 6890 gas chromatograph, 5973 mass selective detector, and 7683 series injector (Agilent Technologies, Palo Alto, CA). Helium flow was 1 mL/min. One μL samples was injected with a split ratio of 1:30 and resolved on a 30 m × 0.25 mm × 0.25 μm ZB-5MSi column (Phenomenex, US). The temperatures for the inlet, interface, and ion source were 230°C, 250°, and 200°C, respectively. After a 5-min solvent delay at 80°C, the oven temperature was increased at 5°C min^-1^ to 310°C at which it was held for 6 min before dropping back to 80°C for the next cycle. Electron impact (70 eV) mass spectra were recorded from m/z 50 to 600 at 2.69 scans sec^-1^. The instrument was autotuned for mass calibration using perfluorotributylamine (PFTBA).

### Data preprocessing

Raw data files (.d) were converted into network common data form (.netCDF) and exported into the Automatic Mass spectral Deconvolution and Identification System (AMDIS_32) for spectral deconvolution (Stein 1999) and database search against the NIST Mass Spectral Database (Rev.D.04.00) and Golm metabolomics library (http://csbdb.mpimp-golm.mpg.de/csbdb/gmd/msri/gmd_msri.html). A list of ion-retention time pairs (IRt) was generated. The IRt data were exported into METabolomics Ion-based Data Extraction Algorithm (MET-IDEA, Broeckling, et al. 2006) for automatic peak alignment, annotation, and integration; Ions were extracted and quantified based on the ion mass/charge (m/z) and retention pairs. The output was generated in Excel format with rows representing different samples and columns representing identified metabolites.

The dataset was then interrogated manually to remove system contaminants, correct annotations if necessary, and reduce uninformative data. Compounds that were identified from library matching with low confidence were eliminated. Compounds identified with high confidence but whose annotation was questionable were labeled with a “?” marker. We also included “known unknown” metabolites if they were found with high confidence. A total of 98 metabolites were identified in forage samples (Additional file 2: Table S2). The intensity value of each metabolite was normalized to both the ribitol internal standard signal and sample dry weight. The resulting data matrix was used for statistical analysis. In an effort to eliminate batch effects, the data matrix was further normalized batch-wise to their respective batch-specific reference sample, such that each metabolite’s intensity value was expressed as a ratio to its value in the appropriate reference sample. The resulting batch corrected data matrix also was subjected to statistical analysis.

### Statistical analysis

The relative standard deviation (RSD% = 100 x standard deviation/mean) for each metabolite was calculated in Microsoft Excel as a measure of the data variability (Parsons et al. 2009; Shurubor et al. 2005).

Principal component analysis (PCA) was performed on the correlation matrix by R statistical package (version 3.0), principal components (PC) 1 and 2 were used to plot the scores.

Hierarchical clustering and heat map was conducted on mean centered and standardized data in R (Version 3.0). Replicate values were averaged where appropriate and Ward’s method on Euclidean distance matrix was used for clustering (Asiago et al., 2012).

## Results and discussion

### Reference samples show batch-to-batch variation

**Figure 1:**
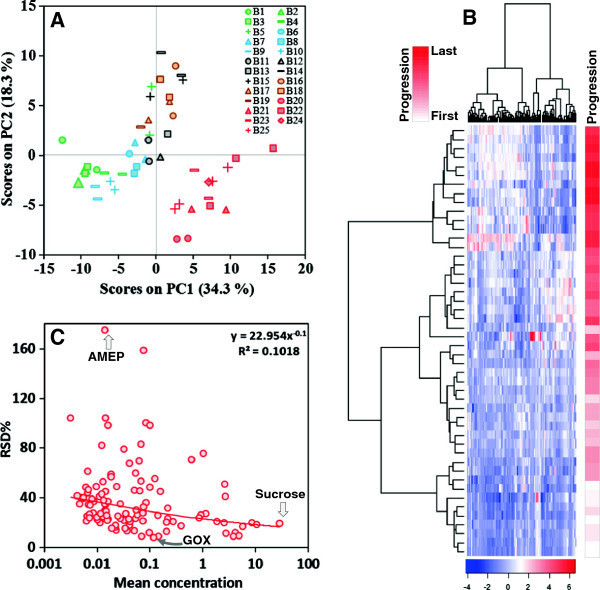
**The reference samples show batch variation.** Same color coding was used to denotes those replicates within the same batch. **(A)** The PCA shows grouping of different batches (B1 to B25) based on the sequence of analysis (green-blue-black-brown-red symbols). The samples analyzed after a new column change form a distinct cluster (red symbols). As expected, replicates within batches mostly cluster together. **(B)** The HCA also shows clustering based on the sequence of analysis (progression: first to last day of analysis). The last ~20% of samples (dark red in progression strips) were analyzed after a column change. Bottom scale bar shows the standardized metabolite levels. **(C)** The RSD values vary considerably among identified forage metabolites, and ranges from 7.8% (for GOX – glucose oxime hexakis (trimethylsilyl)) to as high as 174.9% (for AMEP – 2-Amino-4,6-bis(1,1-dimethylethyl)-phenol). In general, RSD is higher for metabolites at low concentration. Over 86% of the metabolites have their RSD values below 60%. The X-axis is the mean metabolite concentration relative to internal standard. See Additional file 2: Table S2 for the list of metabolites.

To determine the level of batch-to-batch variation, and to identify the possible sources of variation in this GC-MS platform, corn forage reference samples were prepared from the same starting material and analyzed in 26 batches along with other test forage samples (Additional file 1: Table S1). The GC-MS data acquisition spanned approximately two months from beginning to completion (Additional file 1: Table S1). A data matrix was generated from these corn forage reference samples, and was subjected to principal component analysis (PCA). The results indicated that the two technical replicates of reference sample were clustered based on different batches (B1 to B25), suggesting that intra-batch technical variation is marginal (Figure 1A). However, samples also were clustered based on time progression (green-blue-black-brown-red colored symbols) (Figure 1A). Further, samples from B20 to B25 (shown in red) that were analyzed after a column replacement formed a separate cluster from the rest (Figure 1A), suggesting that the column degradation might contribute partially to the batch-to-batch variation.

To further visualize if metabolite quantification was affected by the sequence of analysis across the two-month period, the dataset was subjected to hierarchical cluster analysis (HCA). We observed that the forage reference samples showed clustering based on different batches (day of analysis) and the sequence of analysis (progression: first to last) (Figure 1B). The last ~20% of samples (dark red in progression strips) that were analyzed after a column change all clustered together.

To determine if batch-to-batch variation affects analyte variability in a concentration-dependent manner, we plotted the RSD of all the metabolites against their relative abundance. Approximately 90% of metabolites have RSD values lower than 60%. Only 5% of metabolites had RSD values above 100% (Figure 1C). The plot also reveals an inverse correlation between RSD and metabolite concentration. Abundant metabolites have lower RSD values and low-abundant metabolites have higher RSD values (Figure 1C). However, the correlation coefficient is low (~0.1), and this observation supports the notion that the kind of analytes affects repeatability and reproducibility of a measurement procedure (Linsinger and Josephs 2006).

### Normalization to reference samples reduces systematic variability of the test samples

**Figure 2:**
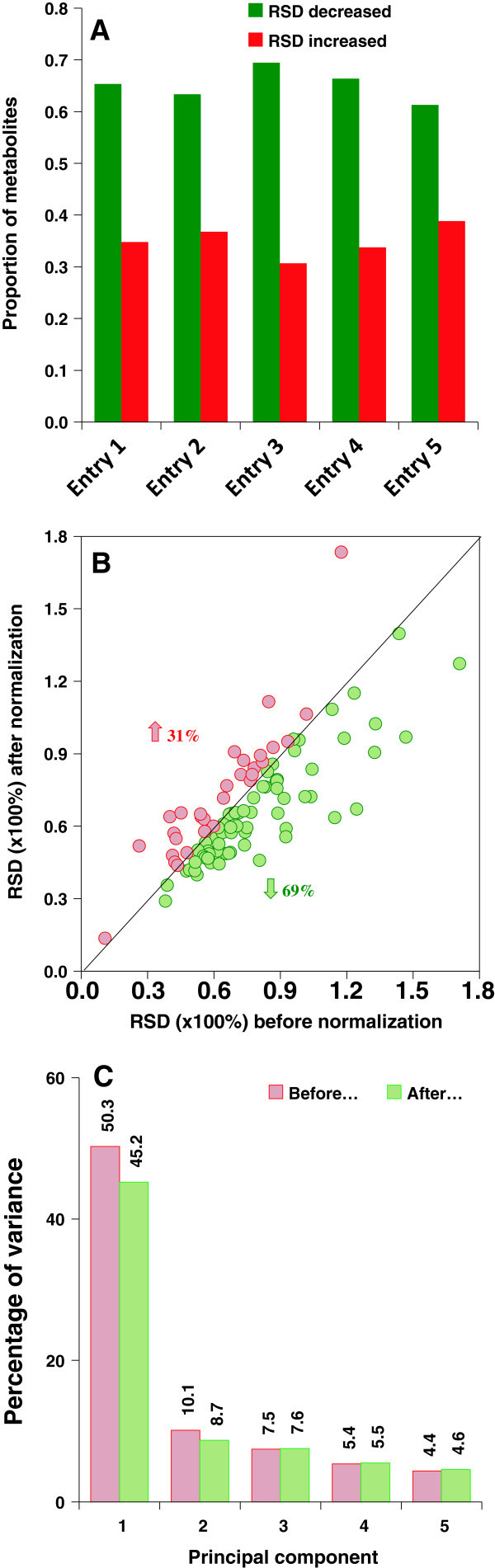
**Data variability of test samples was reduced after normalization against reference samples. (A)** Normalization against reference sample decreases the RSD of over 60% of metabolites in test samples (entries 1-5). **(B)** A scatter plot of RSD for 98 metabolites before and after normalization is shown for entry 3. The green dots (~69% of metabolites, below diagonal line) show decrease and red dots (~31%) show increase in RSD after normalization. **(C)** Normalization against reference sample decreases the percent variance of PC1 and PC2 (in PCA of entries 1-5) indicating that more components contribute after normalization.

As batch-to-batch variation clearly exists in the forage reference samples, it is logical to infer that the test forage samples also had batch-to-batch variation. Since the test samples had similar matrix as the reference sample, it is also reasonable to infer that test samples could behave similarly to the reference sample analyzed within the same batch. Since reference samples were prepared from the same source, theoretically all of them should contain similar amounts of each analyte regardless of batch run. Thus, normalization of the test sample data to the reference sample analyzed within the same batch could potentially correct batch-to-batch variation. To test this possibility, we calculated the relative standard deviation (RSD) for each individual analyte from entry 1 to entry 5 before and after normalization to reference samples. Entries 1 through 5 were selected because they were analyzed in six different batches spanning an 8-week period, and thus potentially have greater batch associated variability. Compared to RSD value before normalization, about two thirds (60-69%) of the metabolites showed a reduced RSD after normalization to reference samples (Figure 2A). However, about one third (31-40%) of the metabolites showed slightly higher RSDs (Figure 2B). Entry 3 showed a slightly higher percentage (69%) of metabolites with reduced RSD after normalization to reference samples compared to other entries (Figure 2A and B).

A PCA was performed on entries 1-5 before and after normalization to reference samples. Since the alteration in PCA score plots is visually not too obvious, we calculated the percentage of variance. We observed that the percentages of variance of principal component 1 and 2 (PC1 and PC2) were reduced after normalization to reference samples (Figure 2C). We also demonstrated that our normalization method affected RSD for each entry in metabolite-dependent manner (Additional file 3: Table S3). This suggests that before normalization to reference samples the observed variance was dominated by a smaller number of metabolites, whereas after normalization more metabolites contributed to the observed variance.

### Advantages of reference sample normalization method

Sangster et al. (2006) first suggested that quality control could be used as a measure to correct batch data variation, and lately this idea was extensively developed (Gika et al. 2007; Dunn et al. 2011, 2012). Thus far quality control is performed by using a normalization standard primarily in two different ways: mean and median correction (Kloet et al. 2009) or LOESS signal correction (QC-RLSC) (Dunn et al. 2011). Usually, the quality control sample is made by pooling small aliquots from each study sample such that it is a representative of the qualitative and quantitative composition of the subject samples being analyzed in the study (Dunn et al. 2012). In this study, we used a bulk corn forage sample to mimic all forage samples, and we termed it as reference sample to differentiate it from quality control samples. Unlike LC-MS, samples for GC-MS analysis require sample derivitization which introduces additional variation. In our method we insert only one reference sample within each batch, and this reference sample was analyzed twice at the middle and end of each batch run. During sample normalization the test sample was normalized to its closest reference samples within same batch to correct within batch variation. In addition, we express each analyte content as a ratio relative to its counterpart in the reference sample such that data from different batches can be directly integrated together. Since each metabolite may differ in stability, derivatization kinetics, as well as instrumental response factors this normalization method essentially takes into account of the uniqueness of each metabolite.

## Conclusions

Metabolomics studies usually involve a large number of samples and require instrumental analysis in multiple batches. Such studies are often aimed to make semi-quantitative measurements of many metabolites of diverse chemical classes in complex sample matrix. It is important to identify platform-specific sources for batch-to-batch data variation, and to minimize or eliminate the batch effect by better experimental design or data pre-processing. GC-MS analysis is prone to systematic error arising from variation in chromatography, ionization, peak integration, derivatization etc. (Styczynski et al. 2007; Kanani and Klapa 2007; Gullberg et al. 2004; Frenzel et al. 2002). In this study we performed PCA of the reference samples that were analyzed in 25 batches, and found that batch-to-batch variation exists in a typical GC-MS platform, but is predictable. The PCA and HCA analysis demonstrated that samples were grouped based on time progression (green-blue-black-brown-red colored symbols) (Figure 1A and B), and suggested that a major source of variation is column degradation due to incremental column bleeding or fouling. This results in gradual tapering of MS signal and ultimately produces the variation from run order and batch effects that were observed (Figure 1). In addition, systematic variation may not be uniform toward all chemical classes; instead this effect may be concentration- and analyte-dependent (Linsinger and Josephs 2006). By normalizating each metabolite individually to those in a reference sample, we demonstrate this method has the potential to correct both within batch and inter-batch data variation, facilitating data integration and statistical analysis.

## Electronic supplementary material

Additional file 1: Table S1: The batch information for corn forage experimental (and reference) sample analysis. (DOCX 39 KB)

Additional file 2: Table S2: The ID, retention time (min), m/z, mean concentration (relative to internal standard), and relative standard deviation RSD%) for 98 metabolites identified from corn forage reference samples. The “?” denotes those metabolites that were detected from the samples with high confidence but their identification were not independently verified. *IDs are based on the order of retention time of metabolites. (DOCX 51 KB)

Additional file 3: Table S3: The RSD value of entry 1 to 5 before and after normalization to reference samples. (XLSX 33 KB)
