# Supplementary material for: A modified data normalization method for GC-MS-based metabolomics to minimize batch variation
Source: Springerplus. 2014 Aug 19;3:439. doi: 10.1186/2193-1801-3-439 (PMC4149678; doi:10.1186/2193-1801-3-439)
Supplement: Supplementary file 1 — Additional file 1: Table S1: The batch information for corn forage experimental (and reference) sample analysis. (DOCX 39 KB) [file 40064_2014_1152_MOESM1_ESM.docx]

**Table S1.** The batch information for corn forage experimental (and reference) sample analysis.

| Batch | Assay date | Test site | Entries |
| --- | --- | --- | --- |
| B1 | 7-11-2012 | Illinois | 1,2,3,4 |
| B2 | 7-12-2013 | Illinois | 4,5,19,20 |
| B3 | 7-13-2013 | Illinois | 20,21,23,24 |
| B4 | 7-14-2013 | Illinois | 25,26,27,29 |
| B5 | 7-16-2013 | Illinois | 29,30,31,33 |
| B6 | 7-18-2012 | Illinois | 33,34,36,38 |
| B7 | 7-20-2012 | Kansas | 1,2,3,4 |
| B8 | 7-21-2012 | Kansas | 4,5,24,25 |
| B9 | 7-22-2012 | Kansas | 25,33,34,35 |
| B10 | 7-23-2012 | Kansas | 36,37,38,39 |
| B11 | 7-25-2012 | Kansas | 39,40,41,43 |
| B12 | 7-26-2012 | Kansas | 43,44 |
| B13 | 7-27-2012 | Kansas | 44,46,50 |
| B14 | 7-28-2012 | Minnesota | 1,2 |
| B15 | 7-29-2012 | Minnesota | 3,4,5,7 |
| B16 | 7-31-2012 | Minnesota | 7,9,10,12 |
| B17 | 8-2-2012 | Minnesota | 12,13,14,15,16 |
| B18 | 8-3-2012 | Minnesota | 16,17,18,19 |
| B19 | 8-4-2012 | Minnesota | 19,20,24,25 |
| New column | | | |
| B20 | 8-17-2012 | Nebraska | 1,2 |
| B21 | 8-20-2012 | Nebraska | 3,4,5,19,20,21,22 |
| B22 | 8-22-2012 | Nebraska | 22,24,25,27,28,29,30,32 |
| B23 | 8-25-2012 | Nebraska | 32,33,34,35,38 |
| B24 | 8-27-2012 | Ontario | 1,2,3,4,5,8,9 |
| B25 | 8-31-2012 | Ontario | 9,10,12,14,16,18,19,20 |
| B26 | 9-8-2012 | Texas | 1,2,3,4,5,24,25,32,33,34,35,  36,39,41,42,43,45,47,48,49 |
