# Supplementary material for: A modified data normalization method for GC-MS-based metabolomics to minimize batch variation
Source: Springerplus. 2014 Aug 19;3:439. doi: 10.1186/2193-1801-3-439 (PMC4149678; doi:10.1186/2193-1801-3-439)
Supplement: Supplementary file 2 — Additional file 2: Table S2: The ID, retention time (min), m/z, mean concentration (relative to internal standard), and relative standard deviation RSD%) for 98 metabolites identified from corn forage reference samples. The “?” denotes those metabolites that were detected from the samples with high confidence but their identification were not independently verified. *IDs are based on the order of retention time of metabolites. (DOCX 51 KB) [file 40064_2014_1152_MOESM2_ESM.docx]

**Table S2.** The ID, retention time (min), m/z, mean concentration (relative to internal standard), and relative standard deviation RSD %) for 98 metabolites identified from corn forage reference samples. The “?” denotes those metabolites that were detected from the samples with high confidence but their identification were not independently verified.

| ID* | Time (min) | m/z | Mean | RSD% | Metabolite name |
| --- | --- | --- | --- | --- | --- |
| 1 | 6.6713 | 64.6 | 0.009763 | 48.66 | Unknown |
| 2 | 6.8958 | 171.1 | 0.027905 | 34.83 | ? 2,4(1H,3H)-Pyrimidinedione, 6-methyl-5-nitro- |
| 3 | 7.1836 | 158.1 | 0.004922 | 40.04 | Isoleucine |
| 4 | 7.597 | 184.1 | 0.054584 | 23.09 | ? Propane, 1-(1-ethoxyethoxy)- |
| 5 | 7.8579 | 221.2 | 1.030741 | 75.35 | Unknown |
| 6 | 10.2136 | 207.1 | 0.012449 | 81.92 | Unknown |
| 7 | 10.888 | 72.1 | 0.008635 | 36.80 | Unknown |
| 8 | 12.7001 | 133.1 | 0.062985 | 69.40 | ? Propanedioic acid |
| 9 | 13.9412 | 283.1 | 0.098821 | 48.10 | Unknown |
| 10 | 15.6501 | 179.1 | 0.142911 | 25.51 | Benzoic acid |
| 11 | 15.7934 | 117.1 | 0.006904 | 34.48 | Unknown |
| 12 | 16.1624 | 116.1 | 0.013918 | 29.30 | L-Serine |
| 13 | 16.3614 | 191.2 | 0.006686 | 61.32 | Unknown |
| 14 | 16.4733 | 174.2 | 0.058568 | 26.03 | Ethanolamine |
| 15 | 16.6272 | 259.1 | 0.006409 | 24.87 | 2-Methyl-3-oxovaleric acid |
| 16 | 16.7523 | 299.1 | 1.158011 | 27.15 | Phosphoric acid |
| 17 | 16.868 | 205.2 | 0.209101 | 12.67 | Glycerol |
| 18 | 17.2421 | 117.1 | 0.008391 | 27.33 | Threonine |
| 19 | 17.4804 | 245.1 | 0.003142 | 104.37 | Maleic acid |
| 20 | 17.5874 | 174.1 | 0.035382 | 26.94 | Glycine |
| 21 | 17.72 | 247.1 | 0.007237 | 26.56 | Succinic acid |
| 22 | 18.1266 | 221.1 | 0.006274 | 21.00 | Unknown |
| 23 | 18.425 | 292.2 | 0.013574 | 22.95 | Glyceric acid |
| 24 | 18.5545 | 75.1 | 0.014398 | 44.89 | Itaconic acid |
| 25 | 18.6245 | 245.1 | 0.014326 | 34.19 | ? 2-Butenedioic acid (E)- |
| 26 | 20.0389 | 184.1 | 0.006723 | 44.00 | ? Methylmaleic acid |
| 27 | 20.6506 | 160.2 | 0.01391 | 34.72 | Aspartic acid |
| 28 | 22.516 | 233.2 | 0.220253 | 23.13 | Malic acid |
| 29 | 23.1071 | 156.1 | 0.075009 | 27.33 | Pyroglutamic acid |
| 30 | 23.1953 | 217.1 | 0.011405 | 19.38 | ? D-glucofuranose |
| 31 | 23.3617 | 174.2 | 0.123171 | 54.97 | 4-Aminobutyric acid |
| 32 | 24.4557 | 292.2 | 0.004236 | 41.20 | Unknown |
| 33 | 26.9022 | 307.3 | 0.007275 | 23.30 | ? 1-Propanol |
| 34 | 27.2362 | 103.1 | 0.022711 | 13.49 | D-Fructose |
| 35 | 27.6634 | 191.1 | 0.008054 | 38.81 | Unknown |
| 36 | 28.2765 | 103.1 | 0.147437 | 8.62 | Xylitol |
| 37 | 28.3922 | 229.2 | 0.156713 | 44.58 | cis-Aconitic acid |
| 38 | 28.4866 | 221.1 | 0.018499 | 58.52 | ? Ethanedioic acid |
| 39 | 28.8989 | 217.1 | 0.014376 | 104.11 | ? Benzene |
| 40 | 29.0309 | 217.2 | 0.041683 | 67.45 | EITTMS_N12C_ATHR_1770.9_1135EC25_ |
| 41 | 29.1334 | 217.2 | 0.004715 | 34.74 | ? 1,3-Dioxolane |
| 42 | 30.0817 | 273.2 | 0.084881 | 36.16 | Citric acid |
| 43 | 30.1343 | 273.2 | 0.068287 | 53.20 | Isocitric acid |
| 44 | 31.0863 | 345.2 | 0.032699 | 21.40 | ? 5-Androsten-3-ol-17-one |
| 45 | 31.4154 | 217.2 | 4.941924 | 9.18 | Fructose, derivative #1 |
| 46 | 31.5186 | 189.1 | 0.076133 | 158.62 | ? 2-Butenoic acid |
| 47 | 31.6193 | 103.1 | 2.819211 | 11.15 | ? 2,4-Bishydroxybutanoic acid |
| 48 | 31.7869 | 319.2 | 0.098354 | 11.52 | Galactose, derivative #1 |
| 49 | 31.9189 | 433.2 | 0.011003 | 40.99 | D-Xylofuranose |
| 50 | 31.9365 | 319.3 | 8.559026 | 19.40 | Glucose |
| 51 | 31.9959 | 133.1 | 0.616348 | 70.44 | Glycerol |
| 52 | 32.1967 | 223.2 | 0.012162 | 43.03 | ? 2-Benzothiophen-4-one |
| 53 | 32.2042 | 205.2 | 2.741903 | 41.07 | Erythrose |
| 54 | 32.2117 | 507.3 | 0.005559 | 39.41 | Benzenamine |
| 55 | 32.5401 | 319.2 | 0.041394 | 17.91 | Mannitol |
| 56 | 32.6789 | 205.2 | 0.209222 | 19.98 | ? 3,8-Dioxa-2,9-disiladecane |
| 57 | 32.6865 | 320.2 | 0.03743 | 13.28 | Vanadium |
| 58 | 32.8303 | 205.2 | 0.064559 | 45.26 | Pyrido[3,4-d]pyrimidin-4(3H)-one |
| 59 | 32.9054 | 217.2 | 0.023588 | 49.01 | ? Idofuranuronic acid |
| 60 | 32.9673 | 191.1 | 0.018671 | 56.58 | Unknown |
| 61 | 33.1199 | 205.1 | 0.012867 | 52.97 | 2,3,4-Trihydroxybutyric acid |
| 62 | 33.3589 | 205.2 | 0.006143 | 48.30 | Pyrido[3,4-d]pyrimidin-4(3H)-one |
| 63 | 33.6904 | 217.1 | 0.005442 | 39.66 | ? Trifluoroacetyl-tris(trimethylsilyl)silane |
| 64 | 33.9087 | 333.2 | 0.040944 | 17.49 | Gluconic acid |
| 65 | 33.9844 | 313.3 | 0.007416 | 34.12 | ? 6H-Dibenzo(b,d)pyran-1-ol |
| 66 | 34.4085 | 204.2 | 0.215882 | 31.64 | EITTMS_N12C_ATHR_2021.5_1135EC44_ |
| 67 | 34.6982 | 333.2 | 0.014739 | 28.90 | Saccharic acid |
| 68 | 35.4719 | 305.2 | 0.388878 | 17.13 | myo-Inositol |
| 69 | 37.2372 | 204.2 | 0.027383 | 22.93 | ? Benzo[1,2-c:3,4-c':5,6-c'']tris[1,2,5]oxadiazole |
| 70 | 37.4861 | 75.1 | 0.006789 | 46.29 | Stearic acid |
| 71 | 39.4172 | 221.1 | 0.014166 | 174.88 | ? Phenol |
| 72 | 39.4747 | 204.1 | 0.041887 | 20.60 | Mannose |
| 73 | 40.4086 | 217.1 | 0.013032 | 24.38 | ? D-mannopyranuronic acid |
| 74 | 42.5823 | 204.2 | 0.04 | 22.63 | Mannose |
| 75 | 44.7054 | 393.3 | 0.016062 | 36.85 | d-Gluconic acid |
| 76 | 44.7129 | 318.3 | 0.054182 | 59.83 | 2-Hexenoic acid |
| 77 | 44.7198 | 364.3 | 0.924739 | 25.99 | ? 1,3,5-Triazine |
| 78 | 44.7304 | 217.2 | 10.85979 | 17.89 | Glucopyranoside |
| 79 | 44.7336 | 319.3 | 1.884848 | 20.82 | Uridine |
| 80 | 44.7579 | 361.3 | 28.7401 | 18.99 | Sucrose |
| 81 | 44.9975 | 204.2 | 0.010307 | 21.89 | Unknown |
| 82 | 45.4798 | 217.2 | 0.022048 | 16.27 | Unknown |
| 83 | 45.6994 | 217.1 | 0.022089 | 17.79 | ? Arabinofuranose |
| 84 | 46.1241 | 204.2 | 0.032735 | 78.99 | Unknown |
| 85 | 46.1942 | 361.2 | 0.077 | 32.31 | ? Melezitose |
| 86 | 46.4394 | 217.1 | 0.051647 | 13.40 | ? Arabinofuranose |
| 87 | 46.5701 | 361.3 | 0.024493 | 25.25 | ? Naringenin-7-O-glucoside |
| 88 | 46.8153 | 217.1 | 0.009923 | 23.84 | Unknown |
| 89 | 47.3558 | 204.1 | 0.0187 | 15.23 | Maltose |
| 90 | 48.7832 | 297.2 | 0.011547 | 26.80 | Benzoic acid |
| 91 | 49.0829 | 204.1 | 0.015521 | 21.42 | Unknown |
| 92 | 49.5251 | 204.2 | 0.033794 | 15.08 | ? L-idopyranuronic acid |
| 93 | 51.3223 | 204.2 | 0.073801 | 20.98 | Mannose |
| 94 | 52.9674 | 221.1 | 0.01597 | 98.11 | Methylcitric acid |
| 95 | 53.9557 | 361.2 | 0.015096 | 25.52 | Unknown |
| 96 | 55.1073 | 217.2 | 0.085495 | 100.30 | Unknown |
| 97 | 55.4808 | 361.2 | 0.099284 | 98.06 | Unknown |
| 98 | 55.5208 | 361.3 | 0.069271 | 82.86 | ? Apigenin-7-O-glucoside |

*IDs are based on the order of retention time of metabolites.
